# Downregulation of lncRNA XR_429159.1 Linked to Brain Metastasis in Patients With Limited-Stage Small-Cell Lung Cancer

**DOI:** 10.3389/fonc.2021.603271

**Published:** 2021-05-17

**Authors:** Ji Li, Wang Jing, Wenxiao Jia, Xiaoyang Zhai, Hui Zhu, Jinming Yu

**Affiliations:** ^1^Department of Oncology, Renmin Hospital of Wuhan University, Wuhan, China; ^2^Department of Radiation Oncology, Shandong Cancer Hospital and Institute, Shandong First Medical University and Shandong Academy of Medical Sciences, Jinan, China

**Keywords:** lncRNA, brain metastasis, small cell lung cancer, risk factors, NET

## Abstract

**Purpose:** The purpose of this study was to identify aberrant long non-coding RNAs (lncRNAs) and explore the predictive value of lncRNA expression patterns on the risk of brain metastases (BMs) in patients with limited-stage small-cell lung cancer (SCLC).

**Patients and Methods:** We executed an array of lncRNA and mRNA chip assays to examine the expression in peripheral blood mononuclear cells obtained from SCLC patients with BMs and compared the expression patterns against those from patients without BMs to identify lncRNAs associated with BMs. Validation was performed against clinical data to further confirm the relationship between lncRNAs and BM. Kaplan–Meier analysis was applied to estimate the cumulative incidence of BMs, and differences between the groups were analyzed using the log-rank test.

**Results:** The expression of 67 lncRNAs (27 upregulated and 40 downregulated) and 47 mRNAs (20 upregulated and 27 downregulated) was significantly different between the BM and non-BM groups (fold change ≥ 2.0, *p*-value ≤ 0.05), based on the lncRNA and mRNA chip assays. Four lncRNAs were verified by quantitative real-time polymerase chain reaction (qRT-PCR) to confirm the accuracy of the microarray data, and the results of 11 patient pairs (11 patients with BM and 11 patients without BM) revealed that low LncRNA XR_429159.1 expression was a high-risk factor for BM. Further clinical data showed that the incidence of BM among 25 patients with low-level LncRNA XR_429159.1 expression was 31% at 1 year, compared with 14.3% among the 18 patients with high-level LncRNA XR_429159.1 expression (*p* = 0.035).

**Conclusion:** Our present study identified the low-level expression of lncRNA XR_429159.1 as a high-risk factor among BM in patients with limited-stage SCLC. LncRNA XR_429159.1 is a critical molecule that regulates SCLC metastasis, involved in the neuroepithelial transforming gene 1 (NET1) pathway, and serum levels of this lncRNA are significantly associated with the risk of BM.

## Introduction

The brain is the most common distant metastasis site for patients with small-cell lung cancer (SCLC), and brain metastasis (BM) is an unfavorable prognosis factor, regardless of the disease stage at presentation. At the time of SCLC diagnosis, ~10–25% of patients suffer from BM, and the probability of BM increases to 50% among patients who survive as long as 2 years after diagnosis (1–3).

The positive role played by prophylactic cranial irradiation (PCI) in patients with SCLC was established by a meta-analysis in 1999, and PCI has become part of the standard treatment modality used in patients with limited-stage SCLC who respond well to initial treatment. However, the role played by PCI has recently been challenged in patients with stage I limited- and extensive-stage SCLC, with fewer survival benefits observed following PCI based on follow-up magnetic resonance imaging study (4–7). Our retrospective study revealed that patients with completely resected p-stage II–III SCLC and lymphovascular invasion were at the highest risk for BM, whereas the incidence of BM among patients with p-stage I SCLC was <10% (8). Therefore, identifying the subgroup with high-risk factors for BM who may obtain a survival benefit from PCI represents an urgent task.

Numerous RNAs are transcribed from the human genome, and protein-coding sequences represent only a small portion of the total transcripts. Non-coding RNAs (ncRNAs) lack protein-coding potential, although some can produce small, functional peptides. LncRNAs were long considered to be transcriptional noise; however, recent evidence suggests that these molecules play important roles in diverse cellular processes, including those associated with normal development and disease pathogenesis. LncRNAs can be divided into two groups according to length: small ncRNAs, shorter than 200 nt, are referred to as microRNAs, whereas longer ncRNAs, >200 nt, are referred to as long non-coding RNAs (lncRNAs). MicroRNAs are a well-characterized group of ncRNAs, able to cause gene silencing effects by regulating messenger RNA (mRNA) degradation and repressing translation repression through RNA interference pathways. In addition, gene expression is more complex when regulated by lncRNAs, often involving multiple mechanisms (9–11). LncRNAs play an oncogenic or a tumor suppressive role. For example, PVT1 is highly correlated in primary human tumors (28). MALAT1 can promote tumorigenesis through the PI3K/AKT pathway (29). PCAT-1 has been implicated in prostate cancer patients (30). In addition, apoptosis induced by anticancer drugs can be reduced PCGEM1 (31). HOTAIR is strongly associated with breast cancer metastasis and patient survival (32). In SCLC, lncRNAs have been correlated with drug resistance, disease progression, and prognosis (12). However, several factors remain unknown regarding the correlation between lncRNA status and BM occurrence in SCLC.

In this study, we used an array of lncRNA and mRNA chip assays to examine the expression levels in peripheral blood mononuclear cells (PBMCs) obtained from SCLC patients to determine the relationships between lncRNAs and BM incidence in patients with SCLC.

## Materials and Methods

### Patients

Total RNA was obtained from six SCLC patients with BM and six patients without BM (four men and two women in each group). Subsequently, 11 pairs of patients were enrolled for the validation arm of the current study between June 2015 and October 2015 at Shandong Cancer Hospital and Institute. All patients were treatment-naive at the time of PBMCs collection. This study was approved by the ethical review board. All patients signed an informed consent approved by the Institutional Review Board.

### RNA Extraction

Peripheral blood was acquired in ethylenediaminetetraacetic acid-containing tubes from all SCLC patients. Human PBMCs were isolated with Ficoll-Hypaque gradients. Total RNA was extracted from PBMCs using TRIzol® reagent (Invitrogen, Grand Island, NY, USA).

### Microarray Imaging and Data Analysis

Total RNAs were labeled for the lncRNA+mRNA Human Gene Expression Microarray V4.0 (4 × 180 K), and each array contained a probe set comprising 40,916 human lncRNA transcripts and 34,235 human mRNAs. The datasets presented in this study have been deposited in the following online repositories under the indicated Accession Number(s) (GEO and GSE161968).

We used a LightCycler 480 PCR System (Roche) to perform quantitative real-time polymerase chain reaction (qRT-PCR), using SYBR Premix Ex Taq™ (TAKARA). Expression data were normalized against the expression of glyceraldehyde 3-phosphate dehydrogenase (GAPDH), and the relative expression levels of each gene were analyzed using the 2^−ΔΔCt^ method.

### Clinical Validation

The peripheral blood, treated with ethylenediaminetetraacetic acid to prevent coagulation, was obtained from 43 patients with limited-stage SCLC and initial radical therapy, without PCI treatment, between January 2016 and November 2016. The expression levels of lncRNA XR_429159.1 were determined, and the patients were divided into the high and low expression groups. BM was the primary endpoint, and all necessary information was acquired by follow-up. The incidence of BM was calculated in each group and compared.

### Statistical Analysis

Statistical analysis was performed using SPSS 16.0 (SPSS Inc.). All data are expressed as mean ± SD. A *p*-value < 0.05 was considered significant. According to the LNCipedia database, all lncRNAs identified in this study have previously been recognized. Kaplan–Meier analysis was used to estimate the actuarial risk of developing BM. A log-rank test was used to compare between the groups. A two-sided *p*-value < 0.05 was considered significant.

## Result

### Identifying the Candidate lncRNAs Associated With BM

**Figure 1:**
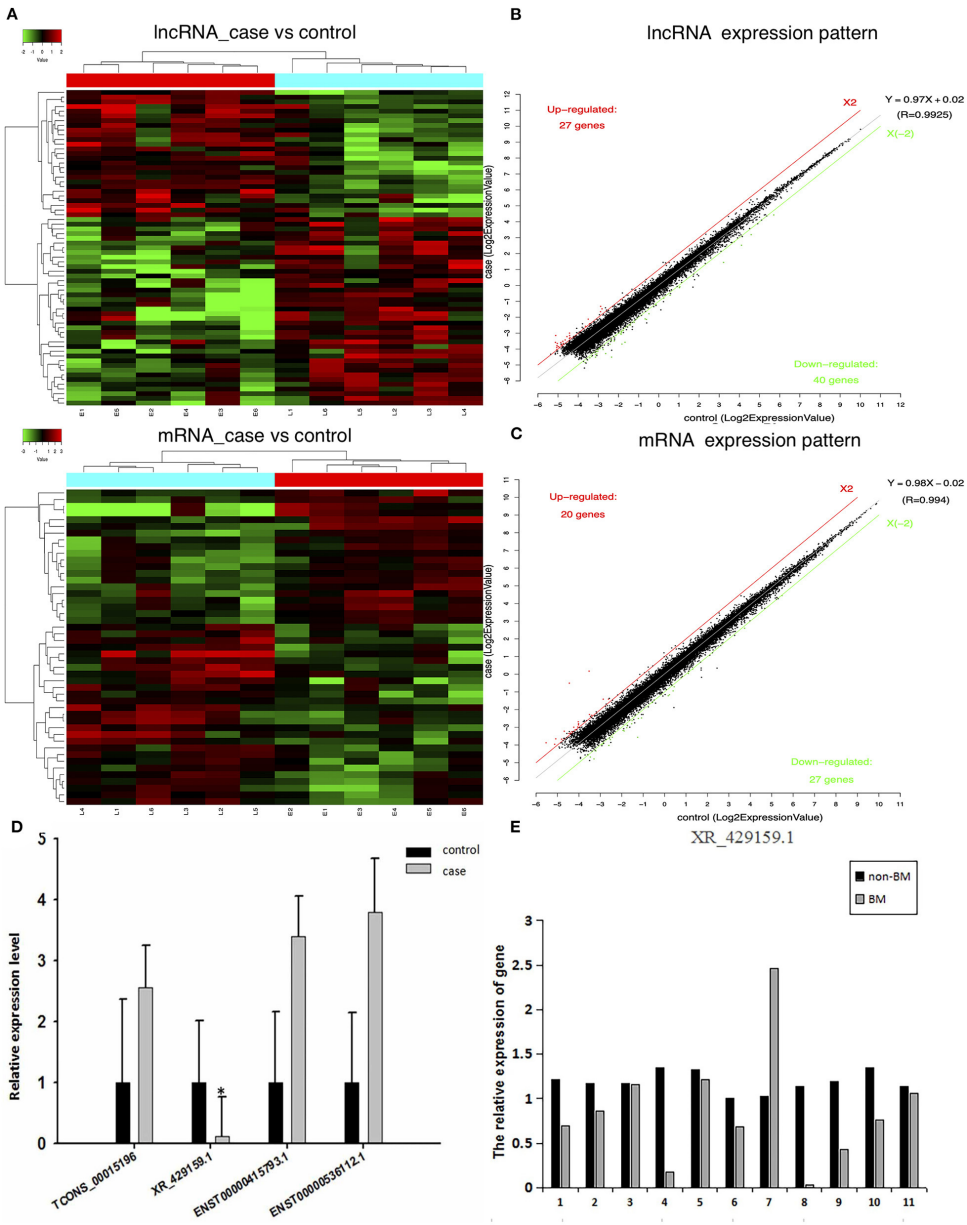
A comprehensive overview of aberrantly expressed lncRNAs and mRNAs in the comparison between BM-SCLC and non-BM-SCLC samples. **(A)** A total of 67 lncRNAs (27 upregulated and 40 downregulated) and 47 mRNAs (20 upregulated and 27 downregulated) were differentially expressed between BM-SCLC (*n* = 6) and SCLC patients without BM (*n* = 6). The scatter plot was used to assess lncRNA **(B)** and mRNA **(C)** expression patterns between BM-SCLC patients and controls. X-axis: log2 expression value. Y-axis: log2 expression value. Red: upregulated. Green: downregulated. **(D)** LncRNA expression levels, as determined by microarray and real-time PCR analyses. **(E)** The comparison between LncRNA XR_429159.1 levels between 11 pairs of patients with and without BM. ^*^p < 0.05.

To confirm the expression levels of lncRNAs and mRNAs in BM-SCLC patients, we performed lncRNA and mRNA microarray analyses on PBMCs obtained from BM-SCLC patients and age- and gender-matched controls (SCLC patients without BM). Cluster 3.0 was used to cluster the lncRNA and mRNA expression data (Figure 1A). Analysis by *t*-test revealed significant differences of up to 2-fold in the expression levels of certain lncRNAs and mRNAs between the groups (*p* < 0.05, Figures 1B,C). As we can see in Figures 1B,C, a total of 67 lncRNAs (27 upregulated and 40 downregulated) and 47 mRNAs (20 upregulated and 27 downregulated) were identified as being differentially expressed in BM-SCLC patients compared with the control patients. The results for the 67 lncRNAs are detailed in Table 1.

**Table 1:** Sixty-seven lncRNAs expressed in BM-SCLC.

**lncRNA ID (up)**	**Probe**	***p***	**lncRNA ID (down)**	**Probe**	***p***
ENST00000443576.2	p4009	0.024	TCONS_00022863	p19374	0.029
ENST00000433357.2	p16736	0.002	XR_109582.2	p25097	0.017
TCONS_00022087	p19097	0.038	ENST00000530576.1	p2682	0.037
ENST00000502776.1	p13887	0.030	TCONS_00008533	p22324	0.017
NR_026881.1	p25115	0.035	XR_429159.1	p42511_v4	0.013
ENST00000442829.1	p9753	0.012	ENST00000509873.1	p12911	0.031
ENST00000547326.1	p3297	0.019	TCONS_00014251	p23618	0.020
TCONS_00015196	p24068	0.004	RNA33685|snoRNA_scaRNA_281_75	RNA33685|snoRNA_scaRNA_281_75	0.016
TCONS_00012509	p23458	0.016	ENST00000547777.1	p3643	0.013
ENST00000569037.1	p6036	0.045	ENST00000599524.1	p8239	0.012
XR_428476.1	p41744_v4	0.001	ENST00000552511.1	p34336_v4	0.021
TCONS_00010550	p22837	0.007	XR_426705.1	p39308_v4	0.045
TCONS_00019442	p18518	0.011	ENST00000536112.1	p34230_v4	0.044
ENST00000441205.1	p9450	0.003	ENST00000596996.1	p8237	0.043
TCONS_00026648	p20573	0.035	ENST00000596705.1	p34609_v4	0.004
ENST00000432973.1	p6336	0.020	ENST00000560026.1	p5053	0.003
ENST00000452503.1	p186	0.018	TCONS_00022023	p19047	0.007
XR_244656.1	p40400_v4	0.000	ENST00000509641.2	p12309	0.046
XR_244697.1	p40446_v4	0.011	ENST00000415793.1	p8562	0.027
TCONS_00024667	p19899	0.027	XR_426704.1	p39309_v4	0.019
uc001ugl.3	p25845	0.039	ENST00000607996.1	p38259_v4	0.029
LIT3591	p28883	0.003	ENST00000434996.1	p11660	0.004
NR_109815.1	p43698_v4	0.003	XR_429229.1	p42634_v4	0.042
TCONS_00013445	p23782	0.005	ENST00000599993.1	p8236	0.018
uc010nlv.1	p26558	0.006	ENST00000416061.1	p17015	0.006
TCONS_00020666	p18986	0.041	ENST00000435749.1	p15383	0.048
ENST00000454882.2	p14463	0.008	ENST00000509350.1	p16341	0.042
			ENST00000598981.1	p29006	0.018
			ENST00000448195.1	p15382	0.018
			ENST00000610044.1	p37042_v4	0.048
			TCONS_00021347	p18641	0.041
			XR_246000.1	p42510_v4	0.008
			TCONS_00014250	p23617	0.008
			TCONS_00022866	p19378	0.026
			ENST00000593653.1	p34611_v4	0.010
			ENST00000607857.1	p38428_v4	0.042
			ENST00000420598.1	p383	0.045
			NR_046095.1	p33675	0.032
			ENST00000459433.1	p38665_v4	0.033
			ENST00000504520.1	p12349	0.009

### lncRNA Expression in BM-SCLC Patients

To verify differences in lncRNA expression between patients with and without BM-SCLC, qRT-PCR was used to verify the up- and downregulation of lncRNA expression between the groups. Based on the potential pathways predicted by the Kyoto Encyclopedia of Genes and Genomes (KEGG) analysis, eight lncRNAs were identified for further validation. Then, four lncRNAs with several differences were selected to confirm the reliability of the microarray data (the forward and reverse primers used for each gene are shown in Table 2). lncRNA XR_429159.1 was validated, demonstrating an 8.33-fold difference in the expression levels between the groups, which was a more considerable difference than the remaining three lncRNAs (Figure 1D).

**Table 2:** The primers of four lncRNAs.

**lncRNA**	**Real-time PCR primers**
lnc196-F2	GGACACAGCAGATAGCAGACC
lnc196-R2	CCACAAGGAAGCCATTGAGACA
lnc159-F1	GCTTGGAGGCTGACAACAAC
lnc159-R1	ACCTGAAGTGAAAAGGAAGAGAAGA
lnc793-F1	AGGAGTGGGCTTTGCTGGAT
lnc793-R1	CTCTAAGTGAAGGAATGTTGTCGTC
lnc112-F2	GGCTTTTAGACCAGGAGACTGTG
lnc112-R2	TCCAGCGAGTGATAGTGGGT

**Figure 2:**
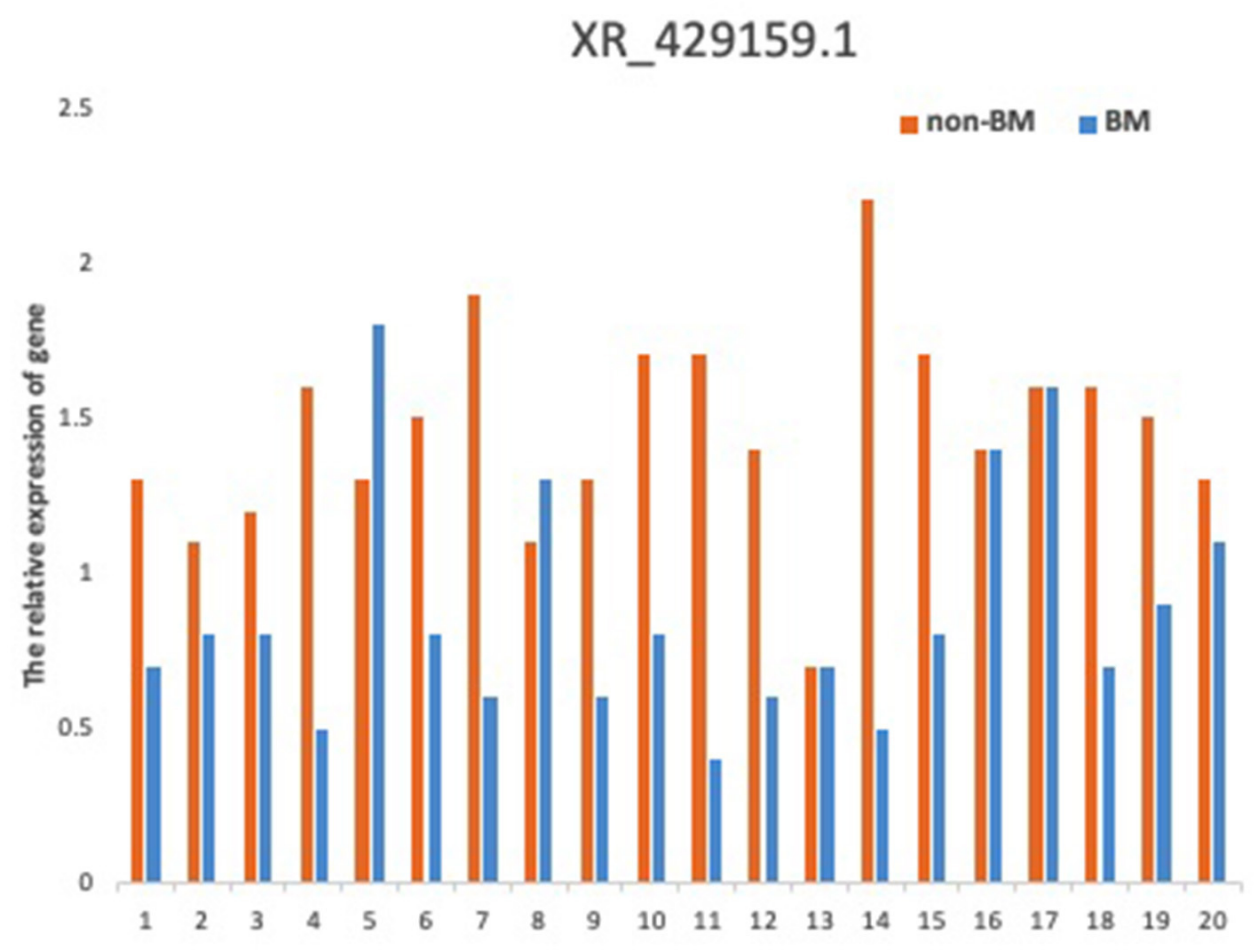
The comparison between LncRNA XR_429159.1 levels between 20 pairs of patients with and without BM.

To verify the relationship between reduced lncRNA XR_429159.1 expression and an increased risk of BM, we collected 11 pairs of patients, matched for clinical data, gender, and age. The results obtained between these patient pairs were consistent with our previous analysis. As shown in Figure 1E, the expression of lncRNA XR_429159.1 was more significantly reduced in patients with BM than in patients without BM, for most pairs, particularly the fourth pair (~8-fold difference in the expression) and the eighth pair (~30-fold difference in the expression). In the seventh pair, the expression level of lncRNA XR_429159.1 was higher in the BM patient than in the control patient; we believe that this may be due to specimen degradation during transport. As shown in Figure 2, to verify this result, we recruited 20 pairs of patients, and the expression of lncRNA XR_429159.1 in 15 patients was more significantly reduced in patients with BM than in patients without BM.

### The Role of lncRNA XR_429159.1 Expression Levels for Predicting BM

**Figure 3:**
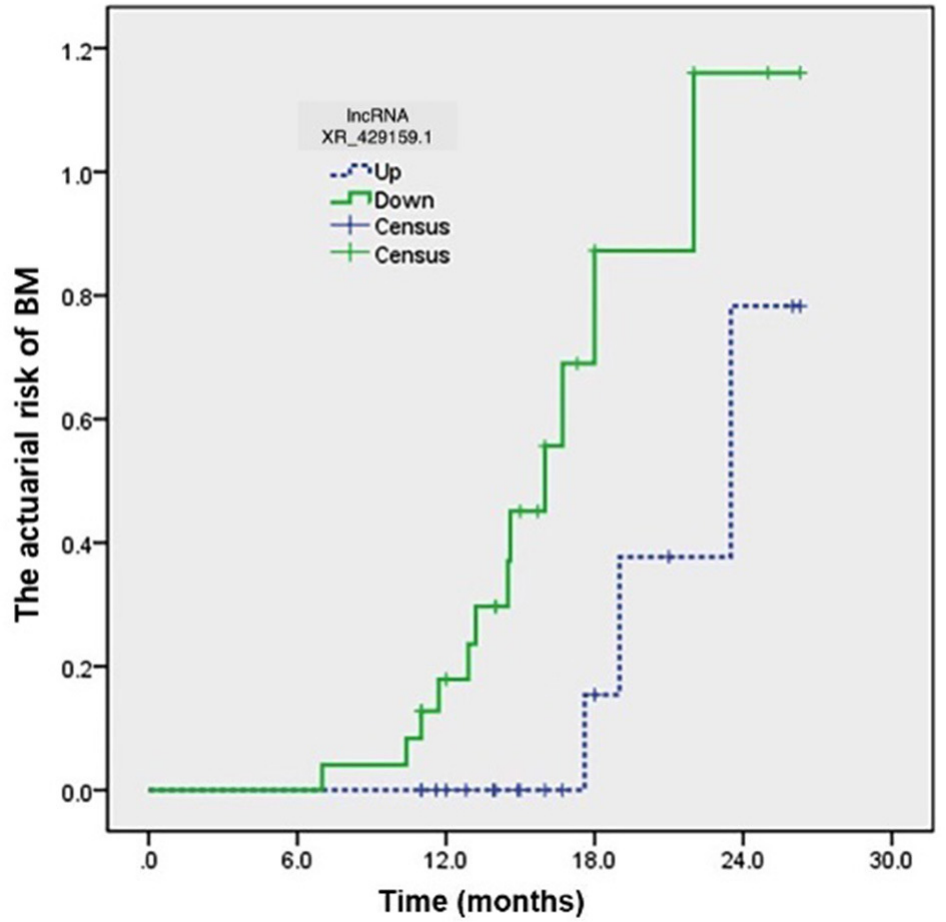
The development time for BM ranged from 7.0 to 26.3 months from the date of diagnosis. The actuarial risk of developing BM at 1 year was 14.3% in patients with high-level lncRNA XR_429159.1 vs. 31.0% in the low-level group. *p* = 0.035.

The clinical characteristics of 43 patients are detailed in Table 3. The last follow-up was conducted in August 2018. Among these 43 patients with limited-stage SCLC, 15 patients (15/43, 34.9%) developed BM. The level of lncRNA XR_429159.1 expression increased in 18 (41.9%) patients and decreased in 25 (58.1%) patients. Three (16.7%) patients developed BM in the high-level group, and 12 (42.9%) patients developed BM in the low-level group. The development time of BM ranged from 7.0 to 26.3 months, with a median time to BM development of 18.0 months, which was determined from the date of diagnosis. The actuarial risk of developing BM at 1 year was 14.3% in patients with high-level lncRNA XR_429159.1 compared with 31.0% in the low-level group, which represented a significant difference, with a *p*-value of 0.035 (Figure 3).

**Table 3:** The characteristics of 43 patients for clinical validation.

	**Total**	**Up (*n*, %)**	**Down (*n*, %)**	***p***
**Age (years)**				
<60	11	5 (27.8)	6 (24.0)	0.779
≥60	32	13 (72.2)	19 (76.0)	
**Gender**				
Male	34	14 (77.8)	20 (80.0)	0.86
Female	9	4 (22.2)	5 (20.0)	
**Smoking status**				
Yes	29	13 (72.2)	12 (48.0)	0.57
No	14	5 (27.8)	13 (52.0)	
**Stage**				
I	12	6 (33.3)	6 (24.0)	0.763
II	25	10 (55.6)	15 (60.0)	
III	6	2 (11.1)	4 (16.0)	
**Treatment**				
S+C/R	17	5 (27.8)	12 (48.0)	0.181
C+R	26	13 (72.2)	13 (52.0)	

### Predictive Signaling Pathway

**Figure 4:**
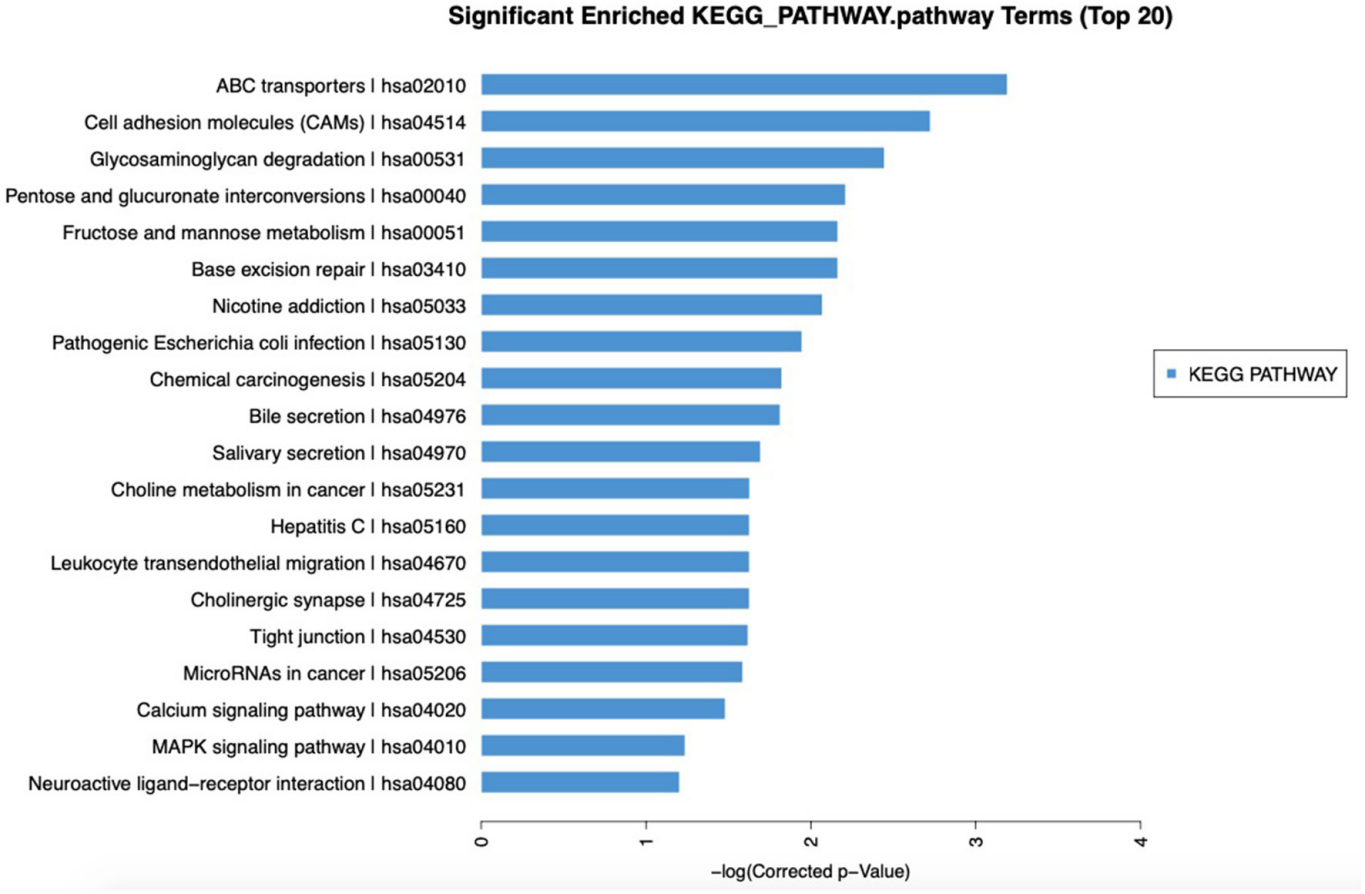
Significantly enriched KEGG pathways. KEGG analysis showed that lncRNA might be related to ABC transporters, cell adhesion molecules, glucose metabolism, and signal transduction pathways.

The relationship between the expression levels of lncRNAs and BM was further explored by identifying the potential signaling pathways associated with lncRNA XR_429159.1 based on analysis using the KEGG signaling pathway database. As shown in Figure 4, KEGG analysis showed that lncRNA might be related to ATP-binding cassette (ABC) transporters, cell adhesion molecules, glucose metabolisms, and signal transduction pathways. We identified all genes 10 kbp upstream and downstream of differentially expressed lncRNAs to identify the potential co-expression target genes and action sites. As shown in Table 4, 15 differentially expressed lncRNAs and co-expressed mRNAs were identified. Comparisons between expression levels in patients with BM and those without BM revealed that the differentially expressed lncRNA XR_429159.1 might be associated with the neuroepithelial transforming gene 1 (NET1) pathway, which may represent a potential target pathway for preventing SCLC BM.

**Table 4:** Predictive signaling pathway.

**lncRNA**	**mRNA**	**Target gene**	**Correlation**	***p***
ENSG00000234773.2	A_33_P3210085	NET1	0.901010299	6.33E−05
ENSG00000232274.1	A_23_P21785	NSUN3	0.921705135	2.03E−05
XR_246000.1	A_33_P3369716	lnc-APBA2-1	0.904952848	5.20E−05
XR_429159.1	A_21_P0011399	NET1	0.926553947	1.49E−05
ENSG00000251301.2	A_33_P3369716	LOC100507195	0.939780957	5.63E−06
XR_246000.1	A_33_P3369716	LOC100507195	0.991972956	2.59E−10
ENSG00000232274.1	A_33_P3361067	ABCG2	0.951285719	1.99E−06
XR_426705.1	A_21_P0010623	LINC01359	0.989767777	8.68E−10
XR_426704.1	A_21_P0010623	LINC01359	0.97878916	3.26E−08
XR_244697.1	A_19_P00320972	STMN1	−0.94305485	4.28E−06
XR_426705.1	A_21_P0010621	LINC01359	0.980554456	2.12E−08
XR_426704.1	A_21_P0010621	LINC01359	0.966813467	3.00E−07
ENSG00000257497.1	A_23_P111662	ABCB5	−0.901917901	6.05E−05
XLOC_007255	A_33_P3339625	IL17C	0.939131971	5.94E−06
ENSG00000233064.2	A_32_P19539	TMEM56	−0.903291397	5.65E−05

## Discussion

In the present study, the genome-wide expression patterns of lncRNAs and mRNAs were examined in PBMCs obtained from six BM-SCLC patients and six controls, and these expression patterns were validated using clinical data. LncRNA XR_429159.1 was significantly downregulated in extensive-stage SCLC patients with BM compared with patients with limited-stage SCLC. The correlation between lncRNA XR_429159.1 expression and BM was further explored in 11 patient pairs, in which limited-stage SCLC patients were matched with extensive-stage SCLC patients with BM. The results showed that a decrease in the expression level of lncRNA XR_429159.1 was associated with the occurrence of BM. To further verify these findings, 43 patients with limited-stage SCLC were closely followed, and 58.1% of the patients were found to have low lncRNA XR_429159.1 expression. The 1-year BM rate for the low expression LncRNA XR_429159.1 group was significantly higher than that for the high expression group (*p* = 0.035). Four patients (≥70 years and stage I) did not receive PCI. Two patients in the low lncRNA XR_429159.1 expression group of these four patients developed BM. We confirmed that low-level lncRNA XR_429159.1 was the most significant risk factor for BM among patients with limited-stage SCLC. Further exploration and analysis suggested that lncRNA may be related to the NET1 pathway. This study may help to identify patients at high risk of BM who may acquire a survival benefit from PCI treatment. Additional studies are planned to reveal the molecular mechanisms and biological functions through which lncRNA XR_429159.1 functions in the pathogenesis of BM-SCLC.

LncRNAs can be found in both the nucleus and cytoplasm, and most lncRNAs located in the nucleus have been found to play important roles in gene expression (13). The abnormal expression of lncRNAs has been associated with the pathogenesis of many diseases, particularly carcinogenesis and the development of malignant tumors (10, 14). Han et al. showed that lncRNA H19 could activate the Janus kinase (JAK)/signal transducer and activator of transcription (STAT) pathway to promote the expression of Stat3 and c-Myc, which play vital roles in the progression of glioblastoma (15). The lncRNA MALAT1 acts through the miR-145-5p/A-kinase anchoring protein 12 (AKAP12) axis to influence prostate cancer therapy (16). Small nucleolar RNA host gene 15 (SNHG15) has been shown to provide a potential therapeutic effect through the regulation of CDK14 protein by sponging miR-486 in non-small-cell lung cancer (NSCLC) patients (17).

Recently, some studies have shown that the abnormal expression of lncRNAs in SCLC may reflect disease progression and predict clinical outcomes. For example, a study indicated that HOXA distal transcript antisense RNA (HOTTIP), which is highly expressed in SCLC tissues, might affect cell proliferation and cell cycle regulation in SCLC patients, promoting SCLC tumorigenesis (18). Another lncRNA, taurine upregulated gene 1 (TUG1), is involved in cell apoptosis, cell cycle regulation, cell proliferation, migration, invasion, and chemoresistance (19). Moreover, TUG1 was upregulated in SCLC tissues and was associated with both the clinical staging and overall survival (OS) of patients with SCLC. Similarly, PVT1, which was overexpressed in SCLC tissues, has also been associated with malignant status and poor prognosis in SCLC patients (20). However, the expression patterns, potential targets, and functions of lncRNAs and their effects on disease development and pathogenesis remain unclear.

LncRNA may affect SCLC through multiple pathways; however, the underlying mechanisms remain unclear. Zhang et al. found that the lncRNA SBF2 antisense RNA 1 (SBF2-AS1) acted as an oncogenic lncRNA in SCLC (21), with increased expression in SCLC cell lines. In addition, high expression of SBF2-AS1 was associated with poor survival, distant metastasis, and lymph node metastasis. Another study indicated that lncRNAs might promote cancer cell stemness in SCLC (22). The overexpression of LncRNA cancer susceptibility 11 (CASC11) increased the proportion of CD133+ cells among SCLC cell lines. Increased cancer stem cells could promote distant metastasis and drug resistance. HOTTIP enhanced chemoresistance in SCLC cells by regulating B-cell lymphoma 2 (BCL-2), increasing the expression of the anti-apoptotic factor BCL-2 and miR-216a (23). In our present study, a low level of lncRNA XR_429159.1 was correlated with BM, further suggesting that the gene regulated by this lncRNA was a suppressor gene.

Because PBMCs are immune cells, we hypothesize that the whole-body immune response can influence BM. Recent studies have shown that immunotherapy is effective for the treatment of SCLC BM. An updated analysis of the CASPIAN trial showed a prolonged, 3.2-month (12.0 vs. 8.8 months) increase in OS in the durvalumab plus platinum-etoposide group compared with that in the platinum-etoposide alone group, with a hazard ratio (HR) of 0.69 (95% CI, 0.35–1.31) (24).

The CASPIAN subgroup analyses examining BM demonstrated for the first time that durvalumab, a programmed death-ligand 1 (PD-L1) inhibitor, can significantly improve OS in SCLC patients with BM. However, few studies have examined the effects of PBMCs on BM. We hypothesized that changes in the regulatory factors associated with PBMCs would lead to changes in immune status, affecting the occurrence of BM. However, because PBMCs are regulated by several factors, the relationships among these factors remain unclear. LncRNA expression was correlated with BM occurrence in some studies (25–27). Therefore, we aimed to explore whether specific molecular markers could be identified in PBMCs that might affect the development of BM, such as lncRNA XR_429159.1.

However, this study has some limitations. Firstly, due to the early development of this study, the included sample size was relatively small, and the subsequent survival data obtained for the clinical sample validation remain immature, which made the impact of lncRNAs on survival challenging to analyze further. Secondly, only preliminary detection and clinical verification have been performed in this study, and no experiments have been performed to further analyze the potential mechanisms through which this lncRNA affects BM, such as its correlation with the NET1 pathway. Thirdly, lncRNAs are diverse and have complex functions. Although some potentially regulated genes were identified based on the second-generation sequencing results, other lncRNAs that may have a potential impact on the development of BM in SCLC cannot be excluded, and only the potential mechanisms of action can be explored based on these predicted results. Therefore, the selection of multiple lncRNAs may help clarify the mechanism of SCLC BM and may provide a reference for clinical drug development.

## Conclusion

In summary, this study described the expression of lncRNAs in patients with BM-SCLC using an RNA microarray method. These findings suggested that SCLC patients with low expression levels of lncRNA XR_429159.1 were at high risk for BM. LncRNA XR_429159.1 is an important molecule that may regulate SCLC metastasis, potentially through the NET pathway, and serum levels of this lncRNA were significantly associated with the risk of BM.

## Data Availability Statement

My data is publicly available, the number and link are GSE161968 and https://www.ncbi.nlm.nih.gov/geo/query/acc.cgi?acc=GSE161968.

## Ethics Statement

This study was approved by The Ethical Review Board of Shandong Cancer Hospital and Institute and complied with the Helsinki declaration. Written informed consent was obtained from all participants.

## Author Contributions

JL and WJ drafted the manuscript. XZ and WJ edited and finalized the manuscript. HZ prepared the figures. All authors contributed to the article and approved the submitted version.

## Conflict of Interest

The authors declare that the research was conducted in the absence of any commercial or financial relationships that could be construed as a potential conflict of interest.
